# The Effect of Physical Impairment Events on the Mental Health of Older Adults

**DOI:** 10.1093/geroni/igaf122.3017

**Published:** 2025-12-31

**Authors:** Bolin Fan, Vivian Lou, Yuting Huang

**Affiliations:** The University of Hong Kong, Hong Kong, China (People’s Republic); The University of Hong Kong, Hong Kong, China (People’s Republic); The University of Hong Kong, Hong Kong, China (People’s Republic)

## Abstract

From a life-cycle perspective, the mental health of older adults is closely related to both past and present physical impairments. Understanding the impact of physical impairment events on the mental health of older adults is crucial for developing effective interventions to improve their life quality and well-being. Using data from the 2015-2018 waves of the China Health and Retirement Longitudinal Study, hypotheses about the relationship between physical impairment events at different stages of life and depression levels and life satisfaction in older adults were tested. The study found that physical impairment events occurring before the age of 50 significantly increased depression levels in older adults but had no significant impact on life satisfaction. Physical impairment events in the past three years had negative effects on both depression and life satisfaction, but the moderating effect of past physical impairments was not significant. These results indicate that both past and recent physical impairment events can lead to negative emotions in older adults, but only recent events can affect life satisfaction. Past physical impairments cannot prepare individuals for recent events. This study provides evidence of the time-dependent impact of physical impairment events on the mental health of older adults, drawing on hedonic adaptation and life course theory. It shows that older adults gradually adapt to physical impairments, with emotional responses regarding life satisfaction decreasing over time. This theoretical contribution highlights the long-term effects of both early and recent health events on mental health and offers valuable insights for improving life-cycle health management services.

